# Corticotropin-Releasing Hormone As the Homeostatic Rheostat of Feto-Maternal Symbiosis and Developmental Programming *In Utero* and Neonatal Life

**DOI:** 10.3389/fendo.2017.00161

**Published:** 2017-07-11

**Authors:** Viridiana Alcántara-Alonso, Pamela Panetta, Patricia de Gortari, Dimitris K. Grammatopoulos

**Affiliations:** ^1^Translational Medicine, Warwick Medical School, Coventry, United Kingdom; ^2^Laboratory of Molecular Neurophysiology, Department of Neurosciences Research, National Institute of Psychiatry Ramón de la Fuente Muñiz, Mexico City, Mexico; ^3^Clinical Biochemistry, Coventry and Warwickshire Pathology Service, UHCW NHS Trust, Coventry, United Kingdom

**Keywords:** corticotropin-releasing hormone, placenta, hypothalamo-pituitary adrenal axis, homeostasis, glucocorticoids, allostasis, neurodevelopment, metabolism

## Abstract

A balanced interaction between the homeostatic mechanisms of mother and the developing organism during pregnancy and in early neonatal life is essential in order to ensure optimal fetal development, ability to respond to various external and internal challenges, protection from adverse programming, and safeguard maternal care availability after parturition. In the majority of pregnancies, this relationship is highly effective resulting in successful outcomes. However, in a number of pathological settings, perturbations of the maternal homeostasis disrupt this symbiosis and initiate adaptive responses with unpredictable outcomes for the fetus or even the neonate. This may lead to development of pathological phenotypes arising from developmental reprogramming involving interaction of genetic, epigenetic, and environmental-driven pathways, sometimes with acute consequences (e.g., growth impairment) and sometimes delayed (e.g., enhanced susceptibility to disease) that last well into adulthood. Most of these adaptive mechanisms are activated and controlled by hormones of the hypothalamo-pituitary adrenal axis under the influence of placental steroid and peptide hormones. In particular, the hypothalamic peptide corticotropin-releasing hormone (CRH) plays a key role in feto-maternal communication by orchestrating and integrating a series of neuroendocrine, immune, metabolic, and behavioral responses. CRH also regulates neural networks involved in maternal behavior and this determines efficiency of maternal care and neonate interactions. This review will summarize our current understanding of CRH actions during the perinatal period, focusing on the physiological roles for both mother and offspring and also how external challenges can alter CRH actions and potentially impact on fetus/neonate health.

## Introduction

The prenatal period represents an immensely challenging phase for the maternal physiology, as it requires continuous adaptive changes to accommodate functional impact of new organs such as the placenta and also increasing and variable demands in response to the new organism’s growth, expansion, and development (1). These changes are also important as they prepare the mother for labor and delivery and later maternal care. A selective redistribution of the fuels used by mother and fetus allows the pregnant mother to preferentially use fat for fuel, preserving the available glucose and amino acids for the fetus and minimizing protein catabolism. This inter-dependent sharing of metabolic resources and nutrient energy substrate availability might influence length of gestation and pregnancy outcomes. In this context, preterm onset of labor may represent a maternal adaptation to limit the metabolic cost of a pregnancy threatened by adverse conditions, or alternatively a fetal adaptation to an unfavorable intrauterine environment.

The intrauterine conditions in which the developing fetus grows have an important role in regulating the function and preserving integrity of its physiological systems later in life. Studies confirm that environmental perturbations altering the intrauterine availability of nutrients, oxygen, and hormones program fetal development and ultimately offspring physiological outcomes (2–11); thus, the ability to mount appropriate adaptive responses and counteract adversity is essential for pregnancy success and to minimize the cost on organism’s health. Lessons from mammalian evolutionary biology and current developmental plasticity concepts suggest that a key player in these pregnancy-induced changes is the hypothalamo-pituitary adrenal (HPA) axis, which regulates central and peripheral homeostatic adaptive responses to stress (12, 13). The HPA axis finely tunes the symbiotic relationship of mother and developing fetus and also determines length of gestation and timing of onset of mammalian parturition. Development of such coordinated responses is crucial since the developing fetus decodes and integrates environmental signals partly via activation of the same biological systems that in mature organisms mediate adaptation and central and peripheral biological and behavioral responses to endogenous and exogenous stressors (e.g., the maternal-placental-fetal neuroendocrine systems). Emerging data also identify the HPA axis as a key player in the development of the progeny especially the first few months outside the intrauterine environment, a critical period for development with considerable interactions between genetics and environment. However, this asymmetric interaction between two organisms in different developmental stages that requires continuous adjustment is poorly understood. This review will examine the current evidence on hormonal responses of the HPA axis initiated by the hypothalamic peptide corticotropin-releasing hormone (CRH) and the mechanisms involved in establishing an effective mother–progeny symbiosis in the prenatal environment and later during the early neonatal period, the effects of which shape the organism’s health and behavior throughout life.

## Maternal HPA Axis and CRH

**Figure 1:**
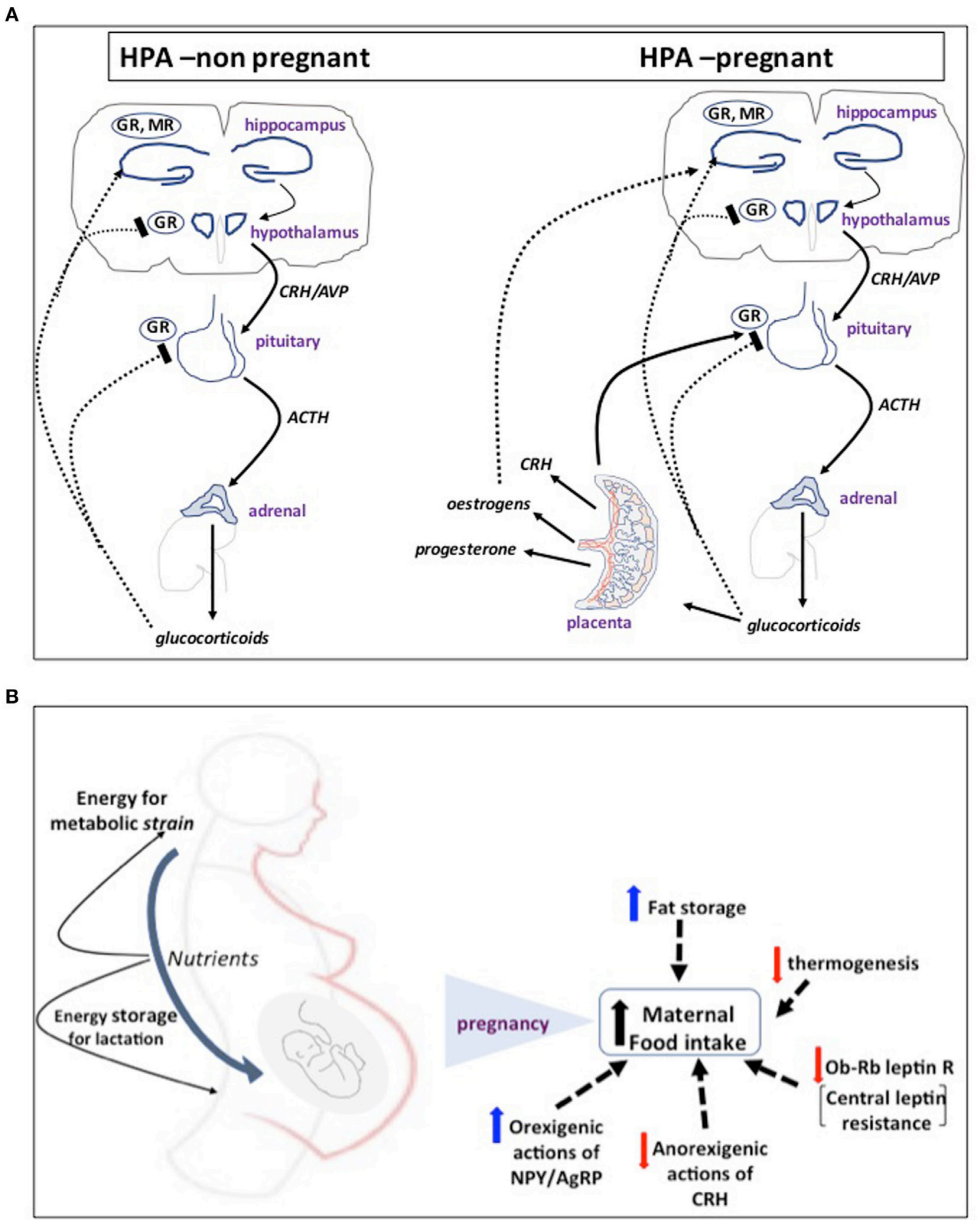
**(A)** The first step of mammalian hypothalamo-pituitary adrenal (HPA) axis activation in response to psychological or physical stressors involves activation of neurosecretory corticotropin-releasing hormone (CRH) neurons in the hypothalamic paraventricular nucleus. CRH and AVP released into the hypophysial portal blood system stimulate adrenocorticotropic hormone (ACTH) release from anterior pituitary corticotrophs and, in turn, the adrenal to secrete glucocorticoids (corticosterone in rodents) into the bloodstream. Cortisol tightly controls HPA activity by feeding back to glucocorticoid receptors (GR) and mineralocorticoid receptors (MR) in the pituitary and hypothalamus to turn-off CRH release and its own secretion. In humans and higher primate pregnancy, placenta secretes large quantities of CRH into the maternal bloodstream during the second and third trimesters of pregnancy. Placental CRH secretion is augmented by cortisol, thus providing a unique positive feedback loop extension to the feto-maternal stress axis that sustains high levels of CRH secretion throughout pregnancy. **(B)** In response to increased energy demands of mother and fetus, food intake increases in pregnancy by resetting central appetite-control mechanisms. A key molecular event appears to be altered leptin signaling and development of central leptin resistance. This is primarily due to decreased expression of the Ob-Rb leptin receptor in the ventromedial hypothalamus and reduced signaling activity despite increased concentration of circulating leptin as a result of expansion of the adipose tissue with contribution from placental secretion. This is coupled with differential regulation of central actions of other orexigenic and anorexigenic hormones and neurosteroids, such as CRH, NPY and AgRP, progesterone, and allopregnanolone that shift the balance toward increased appetite and food intake and fat storage and decreased thermogenesis. These mechanisms ensure that there are sufficient nutrients for the fetus, sufficient energy for the extra metabolic strain on the mother, and a surplus of energy that can be stored as fat in preparation for lactation.

Pregnancy is characterized by dramatic changes in the feto-maternal stress-hormonal system that impacts on the maternal HPA axis function. The anthropoid primate placenta appears to be unique in producing CRH. There are at least two patterns of secretion of placental CRH (pCRH) across gestation among anthropoids. Certain monkeys (*Papio* and *Callithrix*) have an early-to-mid gestational peak, followed by a steady decline to a plateau level, with a possible further rise near parturition. By contrast, humans and great apes placenta secretes large quantities of pCRH into the maternal bloodstream during the second and third trimesters of pregnancy and this exponential rise peaks at parturition (14–17). Interestingly, unlike the hypothalamic peptide, pCRH secretion is augmented by cortisol, thus providing a unique positive feedback loop extension to the feto-maternal stress axis that sustains high levels of CRH secretion throughout pregnancy (Figure 1A). Elevated maternal cortisol at 15 weeks of gestation predicts elevated CRH at 31 weeks, suggesting that elevated cortisol early in pregnancy can exert priming effects on fetal/pCRH system (18).

Both human and rodent feto-maternal tissues have “in-built” defense mechanisms that protect the fetus from exposure to high levels of maternal glucocorticoids and detrimental effects of programming. For example, failure of the 11β-hydroxysteroid dehydrogenase 2 (11β-HSD2) barrier to prevent fetal overexposure is associated with pregnancy complications such as lower birth weight and shorter gestation at delivery (19–21) as well as long-term consequences for the offspring, including resetting of the HPA axis and susceptibility to neurodevelopmental problems and cardio-metabolic disease (22–26).

As pregnancy progresses toward term, maternal hypothalamic production of CRH is downregulated and, thus, the responsiveness of the maternal HPA axis to both physiological and psychological stress is attenuated, a critical adaptation event that provides protection for mother and fetus from the effects of adversity (27–30). It is conceivable that women who fail to show the expected decrease in HPA reactivity to stress and anxiety are at increased risk of complications such as for preterm delivery (31–33), although assessment of anxiety during pregnancy is challenging and can be difficult to distinguish between “normal” pregnancy symptoms, which are common during pregnancy, and atypical somatic complaints such as fatigue, loss of energy, appetite, and sleep changes, which may be related to depression or anxiety.

In the postpartum period, following delivery of the placenta and a sharp drop in pCRH and steroid hormone levels, maternal plasma cortisol levels fall and the function of the mother’s HPA axis and most homeostatic systems gradually return to its pre-pregnant state. Due the prolonged effects of glucocorticoids, the HPA axis is relatively hypo-responsive to dexamethasone suppression for up to 3 weeks postpartum, and recovery of CRH secretion is evident by 12 weeks postpartum (34, 35). Altered HPA responses to pregnancy may influence later maternal mental health and mood disturbances, ultimately compromising quality of maternal care in the postpartum period (36–38).

## CRH–HPA Axis and Metabolic Fuel Distribution Between Mother and Fetus

To meet the increased energy demands of mother and fetus, food intake increases in pregnancy by resetting central appetite-control mechanisms (39), involving development of central leptin resistance and differential regulation of central actions of other orexigenic and anorexigenic hormones and neurosteroids that shift the balance toward increased appetite, food intake and fat storage, and decreased thermogenesis (39, 40) (Figure 1B). These mechanisms ensure that there are sufficient nutrients for the fetus, sufficient energy for the extra metabolic strain on the mother, and a surplus of stored energy. Changes in maternal metabolic state are directly transmitted to the fetus: for example, maternal hyperglycemia in pregnant women induces hyperglycemia and elevated insulin levels in the fetus, thus increasing the risk for complications in infancy and later life (41). Moreover, Barker and colleagues (42) established that derangement of fetal growth trajectories and neonatal development are directly linked to maternal nutritional status, suggesting that both intra- and extra-uterine environment work as a *continuum* to determine the metabolic status of the progeny.

Homeostatic challenges such as prenatal maternal stress can program offspring obesity and metabolic disease risk in adulthood (43–46). This involves pathogenic mechanisms reorganizing immature neural pathways at the hypothalamus (47–49). In this pathological setting, a possible indirect role for CRH in programming homeostasis of energy balance and metabolism at adulthood has been suggested since changes in growth trajectories associated with altered pCRH levels and reduced fetal growth and size at birth are predictors of childhood and adult adiposity (50). In addition, a positive association has been identified between elevated CRH at 30 weeks of gestation and a phenotypic pattern of an early small body size followed by a rapid catch-up growth (51), previously associated with early development of adiposity and insulin resistance (52). Higher circulating levels of CRH also correlate with increased central adiposity (53) and increased levels of adiponectin probably as a compensatory mechanism to improve insulin sensitivity in childhood (54).

Maternal obesity, another type of metabolic challenge, increases risk of fetus developing obesity, insulin resistance, and metabolic syndrome (49, 55–57). It is possible that during maternal obesity, CRH, up-regulated by maternal cortisol, might act as an autocrine/paracrine regulator to increase glucose uptake and facilitate transfer from the mother to the fetus through the upregulation of GLUT1 in the placenta (58). Moreover, CRH could interact with placental and peripheral systems to enhance the production of pro-inflammatory cytokines and pro-inflammatory factors that can reach the fetus. Enhanced secretion from the placenta of cytokines and adipokines are linked to a number of pathological states, including gestational diabetes, hypertension, and intrauterine growth restriction (59).

## CRH–HPA Axis in Fetal Neurodevelopment

**Figure 2:**
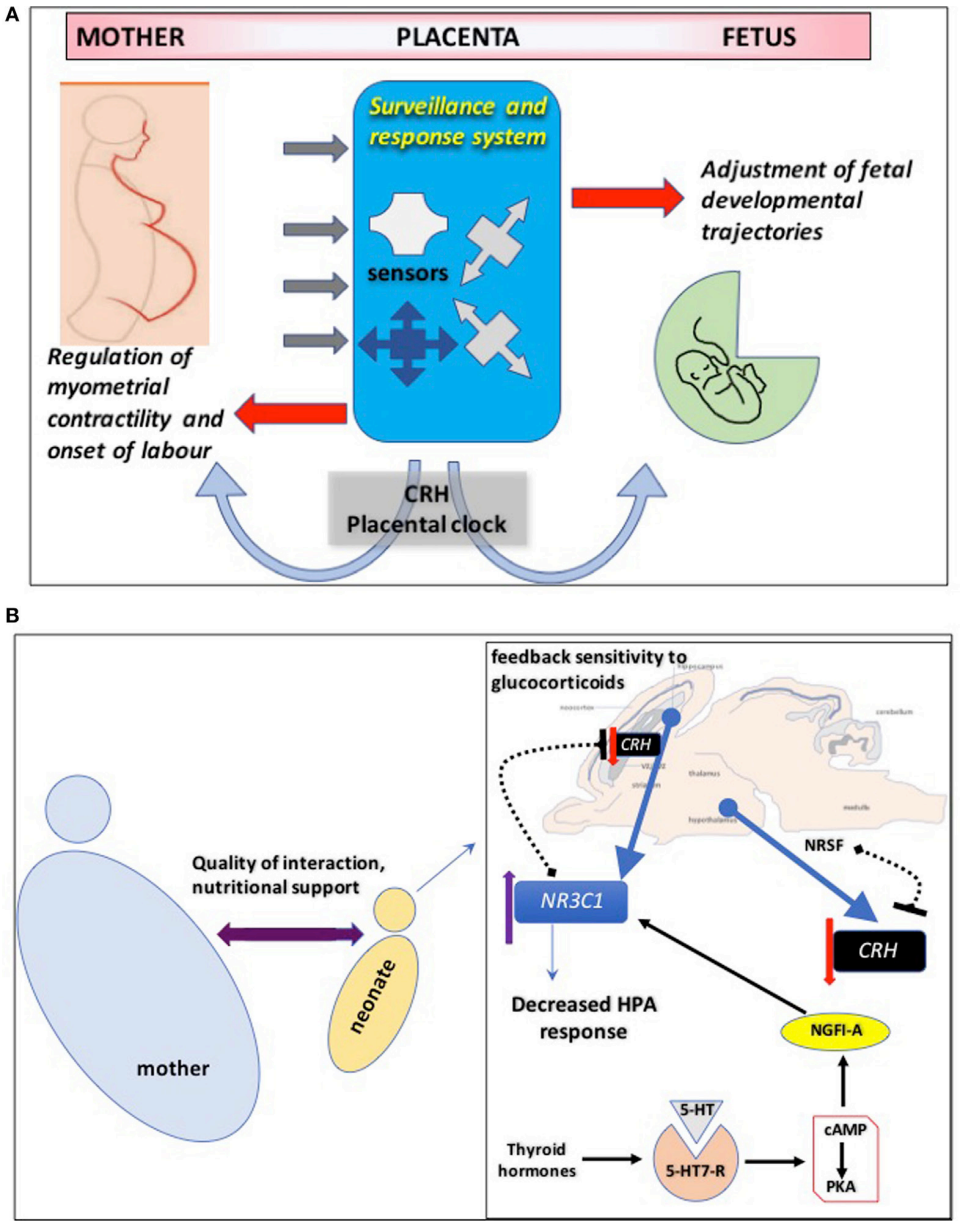
**(A)** Corticotropin-releasing hormone (CRH) controls the placental “surveillance and response” system so that the fetus can detect threats to survival and adjust its developmental trajectory. The placenta employs an intricate network of sensors to decode signals from the maternal environment and prepare the fetus for postnatal survival. Disruption of these sensors might have detrimental effects on fetal neurodevelopment. The “CRH placental clock” might be part of this surveillance and response system: when stress signals such as cortisol from the maternal environment are detected by the fetal/placental unit, the “placental clock” may adapt by altering rate of synthesis of the “master” stress hormone, CRH. The rapid increase in circulating CRH might initiate mechanisms targeting maternal tissues to regulate myometrial contractile machinery and the onset of labor. In parallel, fetal developmental trajectories are adjusted to accelerate maturation of critical organs such as modification of its nervous system to ensure survival in a potentially hostile environment. **(B)** Maternal behaviors resulting in decreased hypothalamo-pituitary adrenal (HPA) axis response to stress in adulthood are most likely driven by enhanced feedback sensitivity of CRH to glucocorticoids in the hippocampus. This involves epigenetic modifications of *NR3C1*, the gene encoding glucocorticoid receptors, in the brain increasing its mRNA expression via mechanisms that promote binding of the transcription factor nerve growth factor-inducible factor A (NGFI-A). This interaction involves a thyroid-hormone-dependent increase in serotonin (5-HT) activity at 5-HT7 receptors and the subsequent activation of cyclic adenosine monophosphate (cAMP) and protein kinase A (PKA) pathway. Hippocampal CRH gene expression is also regulated by maternal care and early life stress through similar epigenetic mechanisms. Similarly, increased histone H3 acetylation and decreased cytosine methylation in the promoter region of CRH gene in hippocampal CA1 layer promote mRNA expression of the neuropeptide. One potential mechanism that enables positive environmental signals (induced by augmented maternal care) to influence transcription of the CRH gene in the offspring hypothalamus involves increased hypothalamic levels of the repressor neuron-restrictive silencer factor (NRSF). NRSF represses the transcription of the CRH gene by binding in the regulatory region of the CRH gene, an epigenetic regulation that persists into adulthood.

Pregnancies complicated by pathological perturbations leading to poor fetal outcomes, including premature labor, hypertension, and intrauterine fetal growth retardation (IUGR) are associated with increased CRH production by the placenta and secretion into maternal blood (60–63). The diverse placental signaling mechanisms regulated by CRH suggest that elevated CRH controls the placental “surveillance and response” system so that the fetus can detect threats to survival and adjust its developmental trajectory (64–67) (Figure 2A). When stress signals (e.g., cortisol) from the maternal environment are detected by the fetoplacental unit, the “placental clock” may adapt by altering rate of synthesis of the “master” stress hormone, CRH. The rapid increase in circulating CRH might initiate mechanisms to regulate myometrial contractile machinery and the onset of labor (68, 69). In parallel, fetal developmental trajectories are adjusted to accelerate maturation of critical organs such as modification of its nervous system to ensure survival in a potentially hostile environment (70, 71).

Evidence point toward delayed fetal maturation and impaired cognitive performance during infancy resulting in decreased brain volume in areas associated with range of cognitive functions, including learning and memory in children (72). These fetal neurobiological adjustments on brain development might involve direct effects of CRH, which can exert neurotoxic effects on hippocampal neurons especially in the immature hippocampus (73–75). In addition, elevated concentrations of CRH may affect directly the developing brain through changes in neuronal sensitivity to CRH actions especially in amygdala and hippocampus, two key regions involved in mediating the central stress response and integrating information regarding “emotional” or “cognitive” stress (76, 77).

The impairment in neurodevelopment associated with altered levels of CRH can be detected early during *in utero* life. Studies assessing neurological integrity during pregnancy suggest that exposure to lower levels of CRH early in gestation may be associated with greater maturity of the fetal central nervous system and accelerated neurological development (70). Moreover, fetal exposure to increased levels of maternal cortisol at 15 weeks and at 19 weeks of gestation and increased levels of CRH at 31 weeks gestation are associated with a significant decrease in newborn physical and neuromuscular maturation. These neonatal measures of maturation reflect altered neurological development detected with magnetic resonance imaging in newborns, including basal ganglia and white matter lesions, as well as motor abnormalities that persist in early childhood (71).

## CRH–HPA Axis and the Interaction Between Mother and the Neonate

After parturition, the connection between neonate and mother shifts to a new physiological equilibrium from a complete physiological dependence to one where two separate individual organisms are in close physical and behavioral contact; this biological relationship can also be influenced by environmental adversity. Young mammals still depend upon the mother for nutrition and physical stimulation as they mature toward complete nutritional independence. Any disruption of the maternal-neonate postnatal interaction by early life events can also induce programming responses with long-lasting consequences. The CRH system is recognized as key for coordinating or even directly inducing adaptations that support the organism to defend against stressful situations such as deterioration of quality of maternal care to maintain homeostasis and survival. However, the immaturity of the CRH system in the newborn makes it vulnerable and susceptible to develop functional abnormalities and ultimately disease later in life (78–80).

In rodents, the period of HPA axis immaturity from birth to the second postnatal week is characterized by a stress hypo-responsive period. This has been attributed mainly to glucocorticoid receptors (GR): reduced functionality in the hypothalamic paraventricular nucleus (PVN) would enhance expression of CRH (81, 82), although enhanced GR negative feedback in the pituitary, would diminish stress response of newborn animals (83). Similar diminished stress response is observed in humans during the first year of life (84) and in non-human primates that also respond to quality of maternal care (85).

Lactation and milk infusion can have beneficial effects on HPA axis activity and maintain low concentrations of circulating corticosterone even in the mother’s absence (80). Acquisition of nutrients during lactation represents a direct bond between infant and mother in early life. Milk composition can also affect various homeostatic parameters, such as growth rate, metabolism, neurodevelopment, and stress reactivity. It is considered as the main vehicle by which glucocorticoids pass from the mother to the offspring (86, 87) and influence the expression of neonatal CRH and ultimately the normal maturation of the HPA axis (88). Thus, maternal diet plays a key role in the type of nutrients that are transferred to the neonate and HPA biological settings; consumption of a high fat diet during pregnancy and/or lactation periods increases not only the fat content of the milk but also the corticosterone plasma levels in dams. This would program the offspring not only toward overweight in the adulthood but also increases corticosterone circulating levels and the risk of metabolic syndrome (89).

## CRH and Metabolic Adjustments Under Postnatal Stress

Disruption of maternal care promotes distinct endocrine, metabolic, and behavioral phenotypic responses in the offspring. Emerging pieces of evidence suggest an early life stress-induced interaction between the HPA axis and energy balance mechanisms leading to deranged metabolic profile later in life associated with insulin resistance. An interesting hypothesis identifies liver 11β-HSD1–HPA axis interactions as a potential pathway in early-life stress-mediated metabolic disturbances, particularly insulin sensitivity, glucose metabolism and lipid synthesis, and mobilization possibly involving changes in glucocorticoid metabolism and signaling (90).

Maternal behaviors resulting in highly nurtured pups that exhibit decreased HPA axis response to stress in adulthood are most likely driven by enhanced feedback sensitivity to glucocorticoids in the hippocampus. This likely involves epigenetic modifications of *NR3C1*, the gene encoding GR, in the brain increasing its mRNA expression (91, 92) (Figure 2B). It is proposed that a global epigenetic signature of early life experience is maintained across species and centered around GR gene regulation, which targets specific regulatory regions such as gene promoters, particularly those of the protocadherin α, β, and γ gene families (93).

By contrast, a disrupted maternal care during the hypo-responsive period could also impact gene expression by opposing epigenetic mechanisms (79, 94). Postmortem studies on human brain samples from suicide victims with reported childhood neglect/abuse support this theory and showed that methylation of the *NR3C1* promoter is increased and expression of GR reduced in hippocampal tissue (95).

Hypothalamic CRH gene expression is also regulated by maternal care and early life stress through similar epigenetic mechanisms (96). Similarly, increased histone H3 acetylation and decreased cytosine methylation in the promoter region of CRH gene in hippocampal CA1 layer promote mRNA expression of the neuropeptide induced by the same stress paradigm (97). Interestingly, environmental enrichment after weaning reverses both the epigenetic upregulation of the CRH expression and the hippocampal synaptic dysfunction of maternally separated animals (97), indicating epigenetic flexibility in this system influenced also by positive stimulus. One potential mechanism that enables positive environmental signals to influence transcription of the CRH gene in the offspring hypothalamus involves the repressor neuron-restrictive silencer factor (98).

A number of maternal deprivation experimental protocols that disrupt maternal-offspring interaction especially during the lactation period and increase the stressful environment of the pups identified altered functionality of the CRH system. These are associated with long-lasting increases in food intake, body weight, and metabolic syndrome risk. Molecular defects identified include decreased expression of CRH-R2 in the ventromedial hypothalamus (VMH) especially in the absence of sensory stimuli (99), a receptor subtype that elicits the anorexigenic effect of CRH and urocortins (100). Lack of maternal care, milk ingestion and hypothermia, lead to increased corticosterone levels (101, 102) and chronically alter the glucocorticoid feedback, maintaining increased synthesis and cerebro-spinal fluid (CSF) concentrations of CRH, as well as basal corticosterone concentrations until adulthood (103, 104). In some examples of impact of neonatal stress in adulthood, adult male offspring show increased CRH expression in the hypothalamus and hippocampus, memory deficits, dendritic atrophy, and altered adult hippocampal neurogenesis (105), whereas female adults show increased corticosterone release in response to stress, anxiety, behavior, and preference to eat palatable food compared to control rats (106). Such a hyperphagic behavior of animals experiencing maternal separation (MS) early in life could be caused by increased hypothalamic expression of NPY (107) associated with a decreased signaling of CRH-R2 receptors in the PVN. Adult MS animals show decreased behavioral and biochemical response to an intra-PVN injection of exogenous urocortin 2, suggesting its altered functionality might be attributable to a chronic stress exposure (108). The levels of CRH-R2 in the VMH are under the control of maternal stimuli in early postnatal life (99), thus, maternal care-driven mechanisms might influence CRH-R2 function in PVN. Similar to animal findings, increased basal glucocorticoid levels, altered glucocorticoid feedback and presence of higher CSF concentrations of CRH are observed in humans subjected to emotional neglect and particularly to parental stress during childhood (109–111). This hyperactivation of the HPA axis system promotes the development of obesity and metabolic syndrome (112, 113) because of the influence of glucocorticoids on orexigenic hypothalamic signals, predominantly stimulating the drive for hypercaloric foods (112, 114, 115). Also, environmental adversity associated with low socioeconomic status during childhood is a predictor of metabolic risk factors, including increased body mass index and altered insulin sensitivity in adults with parental loss and neglect during infancy; all associated with higher basal cortisol levels (79, 115).

## Conclusion

Interactions of the fetus/neonate with the mother are emerging as critical for organism’s development that determines resilience to adversity across the lifespan. In the developing organism, CRH and the HPA axis appear particularly sensitive to exposure to signals from the maternal environment, either physical or behavioral responses to stress or disease. Their ability to influence the activity of molecular switches and mechanisms with key roles in developmental outcomes identifies a critical need to maintain tight homeostatic control of these mechanisms since deranged adaptive responses have devastating consequences either directly or through permanent programming that influence disease susceptibility. Recognizing and characterizing these HPA adaptations (or mal-adaptations) offer a promising strategy for development of novel preventative or therapeutic approaches.

## Author Contributions

All authors (VA-A, PP, PG, and DG) participated in the idea development and equally contributed in the writing up of the manuscript and approved the final version.

## Conflict of Interest Statement

The authors declare absence of any commercial or financial relationship that could be construed as a potential conflict of interest.
